# Immune Defense Mechanism of *Reticulitermes chinensis* Snyder (Blattodea: Isoptera) against *Serratia marcescens* Bizio

**DOI:** 10.3390/insects13030226

**Published:** 2022-02-24

**Authors:** Jian Luo, Zhiqiang Wang, Fang Tang, Kai Feng

**Affiliations:** 1 Co-Innovation Center for Sustainable Forestry in Southern China, Nanjing Forestry University, Nanjing 210037, China; luojian@njfu.edu.cn (J.L.); wzqfafu@163.com (Z.W.); fengkai@njfu.edu.cn (K.F.); 2 College of Forestry, Nanjing Forestry University, Nanjing 210037, China

**Keywords:** cellular immunity, humoral immunity, transcriptome sequencing, quantitative reverse-transcription PCR

## Abstract

**Simple Summary:**

*Reticulitermes chinensis* Snyder is the most important pest in China. *Serratia marcescens* (SM1) can infect insects. In our lab, we found that SM1 can kill *R. chinensis*. However, the mechanisms underlying the immune defense of *R. chinensis* against SM1 is unknown. Therefore, understanding the interaction between *R. chinensis* and SM1 is important for termite control. In this study, immune-related differentially expressed genes (DEGs) in *R. chinensis* were identified and analyzed after SM1 infection. The results increased our understanding of immune responses in pests. This study was helpful for the development of immune suppressive agents in *R. chinensis* management.

**Abstract:**

*Reticulitermes chinensis* Snyder is an important pest species in China. *Serratia marcescens* Bizio (SM1) is a potent biological bacterium. In our lab, we found that SM1 can kill *R. chinensis*. To date, the interaction between *R. chinensis* and SM1 has not been studied. Here, we explored immune responses of *R. chinensis* against SM1 using transcriptome sequencing. To elucidate immune-related genes, we identified 126,153 unigenes from *R. chinensis*. In total, 178 immune-related differentially expressed genes (DEGs) were identified. Kyoto Encyclopedia of Genes and Genomes (KEGG) analysis showed that many cellular responses were enriched in the top 20 terms. Then, we systematically analyzed several cellular immune pathways involved in the response of *R. chinensis* to SM1, including phagocytosis, autophagy, and endocytosis pathways. Furthermore, the expression profiles of the cellular immune-related genes were assessed using quantitative reverse-transcription PCR, and the expression levels of the selected genes were upregulated. Further results revealed SM1-mediated activation of humoral immune responses genes, including Toll, IMD, and melanization pathways, which suggested the involvement of humoral immune responses in the defense against SM1. This research elucidated the mechanisms underlying the immune defense of *R. chinensis* against SM1, providing a solid theoretical basis for exploiting new immune suppressive agents to control *R. chinensis*. Moreover, this study will facilitate the better control of *R. chinensis* using SM1.

## 1. Introduction

Termites are a type of well-known social insects. *Reticulitermes chinensis* Snyder is a lower termite that seriously damages tree xylem tissue and wooden materials used for buildings or furnishings [1,2]. *R. chinensis* is an important pest with concealed activities, small dispersed nests, and strong reproductive capacity [3]. The current methods for the prevention and control of termites mainly include botanical treatments, chemical drug treatments, poison baits, and cultural methods [4,5]. Compared with other methods, biological control methods have many advantages, including efficiency, safety, and durability, and these methods can avoid a series of problems caused by chemical control methods. *Metarhizium anisopliae* has been used to control *Odontotermes obesus* [6]. Wright et al. found that *M. anisopliae* can quickly kill *Coptotermes formosanus* [7]. High concentrations of *Serratia marcescens* and *Pseudomonas aeruginosa* cause high mortality of *Coptotermes curvignathus* [8]. Rhizobacteria can kill *O. obesus* via the production of harmful hydrogen cyanide in vitro [9]. Cyanide from *Pseudomonas fluorescens* can kill *O. obesus* [10]. Fu et al. isolated a bacterium that produces red pigment prodigiosin from dead termite bodies and determined it as *Serratia marcescens* Bizio (SM1) by biochemical experiments and molecular tests [11].

*Serratia marcescens* is a Gram-negative bacterium that produces a bright red pigment [12], and it is widely distributed in plants, animals, water, and soil. In addition, *S. marcescens* generates a series of secondary metabolites [13]. *S. marcescens* has been proven to exert toxic effects on *Microcerotermes championi*, *Heterotermes indicola*, *Bifiditermes beesom,* and *Odontotermes formosanus* [11,12,13,14]. Many studies have proposed that *S. marcescens* is an extremely effective pathogen that can kill pests [15,16,17]. During the process of feeding *R. chinensis* workers in our lab, we found that a number of *R. chinensis* workers died, and their bodies turned red. Ultimately, the laboratory population die out. Therefore, we assumed that SM1 can be used as a pest agent to prevent *R. chinensis*.

When pathogenic microorganisms are used to control harmful insects, insects defend the microorganisms via their immune responses, reducing the efficacy of microbial control. *Wolbachia* and *Spiroplasma* regulated the immune response of *Drosophila melanogaster* to endosymbiotic pathogenic microbes [18]. The humoral and cellular immune responses of insects are involved in defense against infection with pathogenic microorganisms [19]. Therefore, elucidating the molecular mechanism underlying the interactions between microbes and pests will be beneficial for controlling pests.

At present, there has been no report regarding the interaction between SM1 and *R. chinensis*. Therefore, we identified the immune-related genes of *R. chinensis* infected with SM1 using transcriptome sequencing, and the data provided a theoretical foundation for developing strategies to promote immunosuppression in *R. chinensis*. Furthermore, we elucidated the mechanisms underlying cellular and humoral immunity in *R. chinensis* after infection with SM1, and these data provided a theoretical basis for developing more effective biological control methods.

## 2. Experimental Materials and Methods

### 2.1. Insect Rearing and Experimental Treatment

Three different *R. chinensis* nests were obtained from Nanjing Forestry University (Nanjing, China) and were reared in culture dishes at 75% humidity, 25 ± 1 °C, and dark conditions for 6 months. SM1 strain was previously isolated from dead termites and stored in a −80 °C freezer. The SM1 was activated at 30°C for approximately 36 h, and SM1 at a concentration of 1.74 × 10^11^ cells/mL was used for the bioassay. In this study, 20 third-instar workers were taken from three different *R. chinensis* nests and placed in a petri dish (20 cm in diameter) after 12 h of starvation. One microliter of bacterial liquid was placed on the termite pronotum (SM-RC: The *R. chinensis* infected by SM1) (treatment groups), and the control group was administered 1 μL culture medium (RC: The *R. chinensis* treated by culture medium). After 20 h, 10 of the viable *R. chinensis* were directly collected for further sequencing. Three SM1 concentrations (1.74 × 10^11^, 1.74 × 10^10^, and 1.74 × 10^9^ cells/mL) were used to assess the expression of six cellular immunity-related differentially expressed unigenes in *R. chinensis*. Then, 1 μL of bacterial liquid was placed on *R. chinensis* pronotum. Twelve and twenty-four hours after the treatment, the living *R. chinensis* were collected for qRT-PCR. Each treatment was repeated three times independently.

### 2.2. RNA Isolation and Sequencing

Total RNA was extracted from *R. chinensis* using a RNAprep Tissue Kit (TIANGEN, Beijing, China) following the manufacturer’s instructions. The integrity of the RNA was assessed on 1% agarose gels, and the concentration and quality of the total RNA were determined with a NanoDrop spectrophotometer (Thermo Fisher Scientific, Waltham, MA, USA). The messenger RNA (mRNA) was isolated from total RNA by the poly-T magnetic bead method. First-strand cDNA was synthesized with random hexamers, and the second-strand cDNA was synthesized with DNA polymerase I, RNase H, and dNTPs. The cDNA library was sequenced using the Illumina NovaSeq platform by Frassergen Technology (Wuhan, Hubei, China).

### 2.3. Transcript Assembly and Sequence Annotation

The raw data from each sample were processed by filtering low-quality reads and adaptor sequences. Then, high-quality clean data were generated. *De novo* assembly was carried out using Trinity software (https://github.com/trinityrnaseq/trinityrnaseq/, accessed on 15 May 2021), and parameters were set to default parameters [20], and the read quality of the unigenes was evaluated using FastQC software. Assembled unigenes information was stored in FASTA format.

Assembled unigenes were annotated by a BLASTx search. In addition, unigenes were analyzed with NCBI nonredundant protein sequences (Nr) and Swiss-Prot to obtain detailed descriptions. Then, gene functions were classified according to the Gene Ontology (GO) database [21]. Thereafter, the annotation of the unigenes was performed against euKaryotic Ortholog Groups (KOG) and Kyoto Encyclopedia of Genes and Genomes (KEGG) [22,23].

### 2.4. Differential Gene Expression between R. chinensis Infected with SM1 and the Control R. chinensis

To reveal the mechanisms underlying the immune response of *R. chinensis* to SM1, we tried to identify immune genes whose expression in *R. chinensis* was altered in response to SM1 using Illumina NovaSeq sequencing.

The obtained reads were mapped on each unigene using Bowtie2, and the differential gene expression based on fragments per kilobase per million bases (FPKM) was analyzed on DESeq2 (an R language package). To identify the differentially expressed genes (DEGs) between healthy *R. chinensis* and *R. chinensis* infected with SM1, a false discovery rate (FDR) < 0.05 and an absolute value of log2FoldChange > 1 or log2 FoldChange < −1 (FoldChange, (treatment/control) for one gene) were used. Subsequently, GO annotation and KEGG enrichment were used to analyze the DEGs in different processes or pathways.

### 2.5. Quantitative Reverse-Transcription PCR (qRT-PCR)

Total RNA was extracted from six *R. chinensis* workers using TRIzol reagent (Invitrogen, Thermo Fisher Scientific, Waltham, MA, USA) according to the manufacturer’s protocol. First-strand complementary DNA (cDNA) was synthesized with the PrimeScript RT Reagent Kit for qRT-PCR (Takara, Dalian, China) using 1000 ng of total RNA. The online software Integrated DNA Technologies (IDT) (https://sg.idtdna.com, accessed on 5 October 2021) was used to design gene-specific primers for qRT-PCR based on the unigene sequences (Table S1). The internal reference genes were β-actin and GAPDH. The 20 µL volume for qRT-PCR (TB Green Fast qPCR Mix) (Takara, Dalian, China) contained 5 ng/µL template cDNA (the final concentration), 10 µL of 2 × SYBR Premix Ex Taq, 0.4 µL of 50 × ROX Reference Dye II, 0.2 µM forward and reverse primers (the final concentration), and 7.8 µL of double-distilled water. The detection instrument was an ABI ViiATM 7 Real-time PCR system (Applied Biosystems, Foster City, CA, USA). To determine the amplification efficiency of each gene, LinReg PCR software (version: September 2014) was used to examine the experimental results. Each sample was analyzed three times independently. The 2^−ΔΔCt^ method was used to calculate the relative mRNA expression of genes in *R. chinensis* [24,25].

### 2.6. Statistical Method

InStat software (GraphPad, San Diego, CA, USA) was used to analyze the variance of the experimental data. Student’s t-test was used to compare two samples (treatment and control), and *p* < 0.05 (*) and *p* < 0.01 (**) indicated that the data were statistically significantly different between the treatment and the control groups. One-way analysis of variance (ANOVA) and Tukey’s multiple comparisons were used to assess statistical significance among multiple samples, and *p* < 0.05 indicated that the data were statistically significantly different.

## 3. Results

### 3.1. Analysis of R. chinensis Transcriptome Data

#### 3.1.1. *R.* *chinensis* Transcriptome Assembly

Following the removal of low-quality reads, 30,728,402–33,572,113 clean read pairs were obtained for the RC libraries. In addition, 28,939,264–30,947,431 clean read pairs were yielded for the SM-RC libraries. The quality and throughput of the transcriptome data were listed in Table S2. Subsequently, the clean reads were assembled by Trinity software, and 126,153 unigenes with sizes in the range of 500–2000 bp were obtained from clean read pairs with a N50 length of 1088 bp (Table 1). Following the removal of short-length and repeated sequences from the *R. chinensis* transcriptomes of the control group and treatment group, 394,290 Nr unigenes were annotated by BLAST searches in the following databases: KOG, KEGG, NR, GO, and Swiss-Prot. According to the results, 17.50, 19.90, 30.07, 15.23, and 25.78% of the unigenes were annotated in KOG, KEGG, NR, GO, and Swiss-Prot, respectively (Table S3).

#### 3.1.2. Functional Annotation of *R.* *chinensis* Transcripts

The GO database was used for gene functional annotation using Blast2GO software. The unigenes of *R. chinensis* from transcriptome sequencing were classified into three GO functional processes, including biological processes, cellular components, and molecular functions. These unigenes were distributed in 59 GO terms (Figure S1). To further verify the reliability and integrity of the annotation process, 68,985 unigenes were successfully mapped to the KOG categories, which comprise 26 categories (Figure S2). The top three subcategories were posttranslational modification, protein turnover and chaperones, general function prediction, and signal transduction mechanisms. In this study, increased attention was provided to the function of signal transduction mechanisms in the *R. chinensis* immune response to SM1. To thoroughly understand the biological pathways activated in *R. chinensis* infected with SM1, 78,454 unigenes were mapped to 34 main pathways (Figure S3). Notably, 6871 unigenes were mapped to the immune system.

#### 3.1.3. DEGs in the Response of *R. chinensis* to SM1 Infection

**Figure 1:**
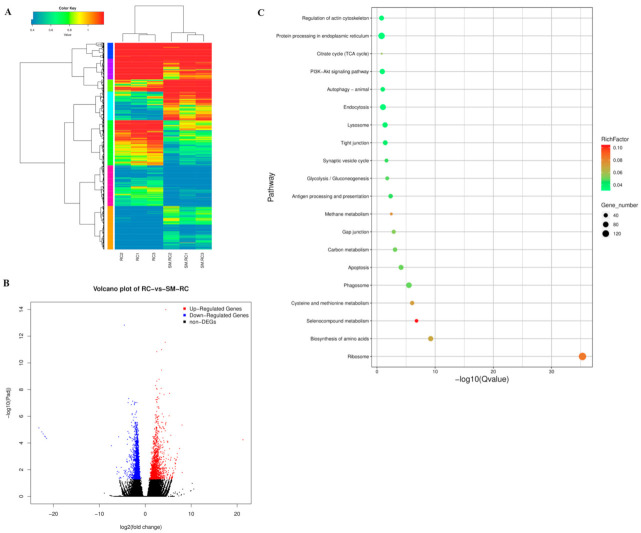
Overview of DEGs. (**A**) Heatmaps illustrating differences in normalized log signal intensities of the identified genes in *R. chinensis*. RC1, RC2, and RC3 represent the control group. SM-RC1, SM-RC2, and SM-RC3 represent the treatment group. Red bars indicate genes expressed at high levels, and blue bars indicate genes expressed at low levels. Color changes from blue to red indicate gradually increasing expression. (**B**) Volcano plots showing the DEGs between SM-RC and RC. Blue and red spots indicate significantly downregulated and upregulated genes, respectively. Black dots represent genes that are not differentially expressed. (**C**) The top 20 KEGG enrichments for differentially expressed unigenes in *R. chinensis*. The vertical axis indicates the name of the pathway, the names are sorted by Q value from low to high, and the horizontal axis indicates −log10 (Q value). The size and color of the dots indicate the number of DEGs and the corresponding RichFactor ranges, respectively.

The DEGs between SM-RC and RC were identified by the DESeq2 package. A total of 3,037 DEGs were identified with a threshold of FDR < 0.05 and |log2FC| > 1 (Figure 1A). Among these DEGs, 2435 DEGs were mapped to KEGG pathways (Supporting Information Figure S4). We found that 1707 DEGs were downregulated and 1,330 DEGs were upregulated (Figure 1B). For the KEGG pathways, the top 20 enriched KEGG terms in this study (Figure 1C) included endocytosis, lysosome, autophagy, and phagosome immune pathways.

### 3.2. Verification of Transcriptomic Data by qRT-PCR

**Figure 2:**
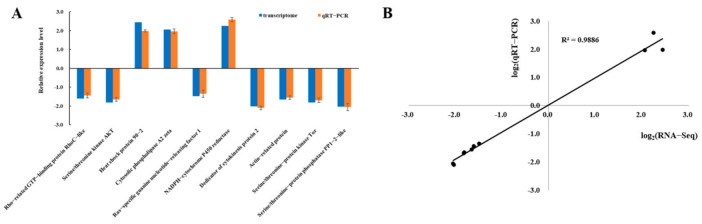
Verification of the expression of 10 selected differentially expressed unigenes by qRT-PCR. (**A**) The expression levels of the selected unigenes were measured using qRT-PCR. The qRT-PCR and transcriptome columns represent the mean average delta–delta Ct values and the log2FoldChange value, respectively. (**B**) The scatter plots show the R-squared (R^2^) and linear regression between the transcriptome data and qRT-PCR values.

To verify the expression pattern of differentially expressed unigenes identified in the transcriptome library, 10 differentially expressed unigenes were selected and their expression levels were quantified using qRT-PCR. β-actin and GAPDH were used as an internal reference for qRT-PCR normalization. The results showed that the expression trend of the selected unigenes was consistent with the sequencing data (Figure 2A). In addition, the correlation between the qRT-PCR results and sequencing data was assessed, and the coefficient of correlation (R^2^) was 0.9886 (Figure 2B). In summary, the qRT-PCR results proved the reliability of the expression profiles observed in the transcriptome data.

### 3.3. Immune-Related DEGs in R. chinensis Infected with SM1

**Figure 3:**
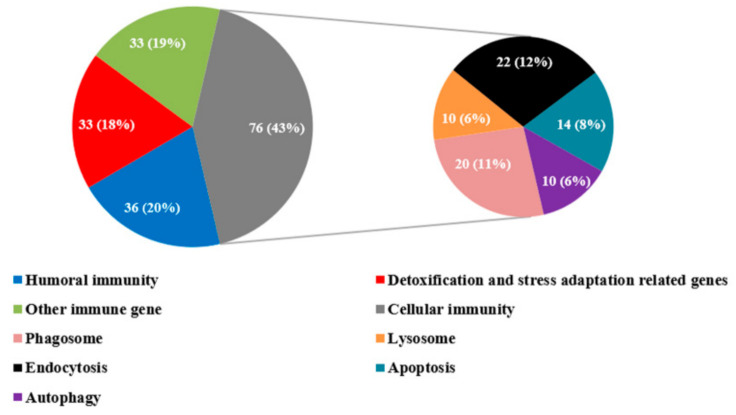
The distribution of immune-related DEGs in the categories of humoral immune, detoxification and stress adaptation-related genes, cellular immunity (phagocytosis, endocytosis, apoptosis, and autophagy)-related genes, and other immune-related genes. The number (percentage) of each category of immune-related genes are shown in each pie chart. All of the mentioned genes are shown in the context and Table S4.

We identified immune-related DEGs with the sequencing data and qRT-PCR analysis. A total of 178 immune-related DEGs were manually identified from all of the annotated unigenes by NCBI Blast and a comparison of known sequences from other species (Table S4). These immune-related DEGs were classified into four categories: Genes related to cellular immunity, humoral immunity, genes related to detoxification and stress adaptation, and other immune-related genes (Figure 3).

Regarding cellular responses, 76 DEGs in *R. chinensis* were divided into endocytosis, phagocytosis, apoptosis, and autophagy (Figure 3). We hypothesize that cellular immunity may play an important role in this study. For humoral immunity, pattern recognition proteins, signaling transduction genes (including the immune deficiency (IMD) pathway, Toll pathway, JAK/STAT pathways, and melanization pathway), and effector genes were identified by combining sequencing data and qRT-PCR. For detoxification and stress adaptation-related genes, cytochrome P450s and heat shocked proteins were identified from our sequencing data. Many other immune-related genes, such as ATP-binding cassette subfamily genes and Rac 1-related protein were identified from our sequencing data. In summary, the immune system of *R. chinensis* was activated after an infection with SM1. To better understand the immunity of *R. chinensis*, we attempted to reveal the immune reactions that occur in *R. chinensis* by carrying out the following analysis.

#### 3.3.1. Cellular Immune Responses Induced by SM1 in *R. chinensis*

**Figure 4:**
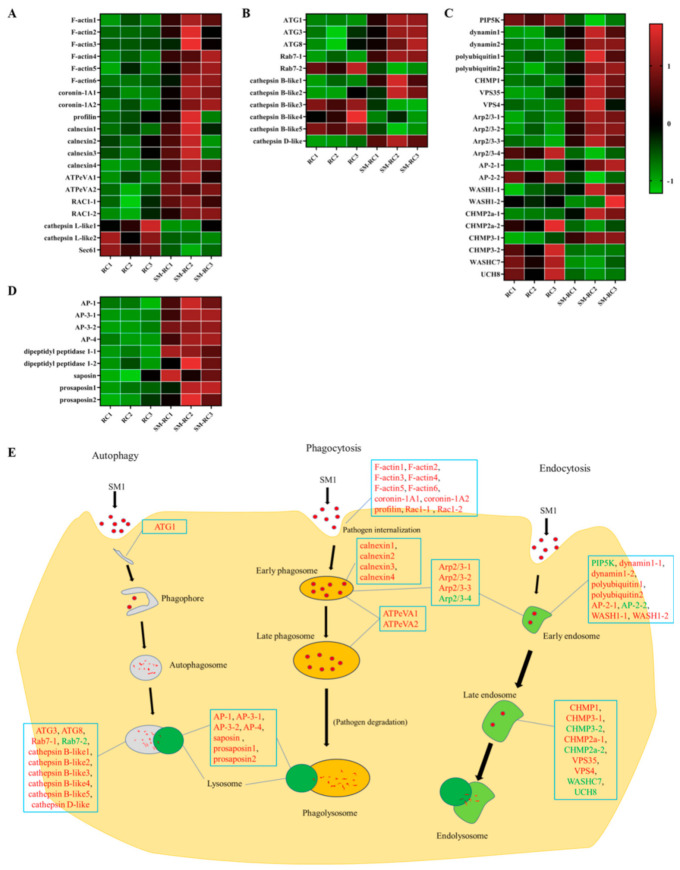
DEGs involved in the cellular response of *R. chinensis* infected by SM1. (**A**) Heatmap analysis of phagocytosis-related genes. (**B**) Heatmap analysis of autophagy-related genes. (**C**) Heatmap analysis of endocytosis-related genes. (**D**) Heatmap analysis of lysosome-related genes. (**E**) Schematic overview of a putative model of the cellular immune responses that occur in *R. chinensis* infected with SM1. RC1, RC2, and RC3: The control groups. SM-RC1, SM-RC2, and SM-RC3: *R. chinensis* infected with SM1.

Several cellular immune pathways were enriched in the top 20 KEGG pathways, including phagosomes, autophagy, and lysosomes (Figure 1). The phagosome enrichment factor was higher than other cellular immune pathways, and many differentially expressed genes were identified in *R. chinensis* infected with SM1 (Figure 4). Coronin-1A can regulate actin network dynamics and mediate phagocytosis in insects. Moreover, profilin regulates the progression of phagocytosis and the high expression of F-actin is initiated by phagocytosis. The expression levels of two coronin-1A genes, six F-actin genes, and a profilin gene were upregulated when *R. chinensis* was infected with SM1 (Figure 4A). V-type proton ATPase catalytic subunit A (ATPeVA) is important for phagocytosis, and two ATPeVAs are upregulated in *R. chinensis* infected by SM1 (Figure 4A). Calnexin is important for phagocytosis in the endoplasmic reticulum. In our sequencing data, we found that the expression levels of three calnexins were upregulated in *R. chinensis* infected with SM1. Ras-related C3 botulinum toxin substrate 1 (Rac1) is involved in the regulation of phagocytosis, and the expression of two Rac1 was induced by SM1 in *R. chinensis*. The expression levels of two cathepsin L-like genes and one transport protein Sec61, which are component genes related to phagocytosis, were downregulated (Figure 4A). In this paper, these DEGs indicate that the internalization of phagocytotic internalization by *R. chinensis* initiates resistance to SM1 infection.

Autophagy is an evolutionarily conserved process used to eliminate pathogens in insects. Three autophagy-related genes (ATGs) were identified in the sequencing data. The expression levels of ATG1, ATG3, and ATG8 were upregulated in *R. chinensis* after infection with SM1 (Figure 4B). The expression of two Ras-related protein Rab-7a-like (Rab7) genes were differentially expressed in *R. chinensis* (Figure 4B), which is conducive to the fusion of autophagosomes and lysosomes. Moreover, we found that cathepsin D-like and cathepsin B-like proteinases in this pathway were differentially expressed. These results suggest that autophagy participates in defending *R. chinensis* against SM1.

Endocytosis plays a vital role in cellular immunity, including the dual role of surveillance and elimination of foreign pathogens. Dynamin can mediate the fission of the plasma membrane and promote the formation of the clathrin triskelion lattice. Moreover, low-density phosphatidylinositol-4-phosphate 5-kinase family protein (PIP5K) initiates the endocytosis pathway. We found that the expression levels of two dynamins were upregulated and that the expression level of PIP5K was downregulated in *R. chinensis* (Figure 4C). The expression levels of some regulator genes in this pathway were upregulated, including polyubiquitin, charged multivesicular body protein 1a like (CHMP1), vacuolar protein sorting-associated protein 4B-like (VPS4), and vacuolar protein sorting-associated protein 35 (VPS35) (Figure 4C). Several genes were differentially expressed in *R. chinensis* after SM1 infection, including actin-related protein 2/3 complex (Arp2/3), AP-2 complex subunit alpha (AP-2), WAS protein family homolog 1-like (WASH1), charged multivesicular body protein 2a like (CHMP2a), charged multivesicular body protein 3 (CHMP3), WASH complex subunit 7-like (WASHC7), and ubiquitin carboxyl-terminal hydrolase 8-like (UCH8) (Figure 4C). Here, we hypothesize that endocytosis of *R. chinensis* was activated by SM1.

Lysosomes are important components that degrade extracellular threats. The expression levels of three kinds of adaptin-like proteins (AP1, AP3, and AP4) were upregulated in *R. chinensis* by SM1 (Figure 4D). The expression levels of two dipeptidyl peptidases that function as transmembrane glycoproteins in immune cells were upregulated in *R. chinensis* (Figure 4D). The expression of saposin and two prosaposins was upregulated and these proteins are involved in the lysosome pathway. These DEGs related to lysosomes indicate that the lysosome pathway was activated, and phagosomes, endosomes, and autophagosomes may have fused with lysosomes.

In summary, these cellular immune pathways of *R. chinensis* played vital roles in defending SM1. The regulatory mode of cellular immune pathways is shown in Figure 4E.

#### 3.3.2. Expression Profiles of Cellular Immunity-related Genes in *R. chinensis* Infected with SM1

**Figure 5:**
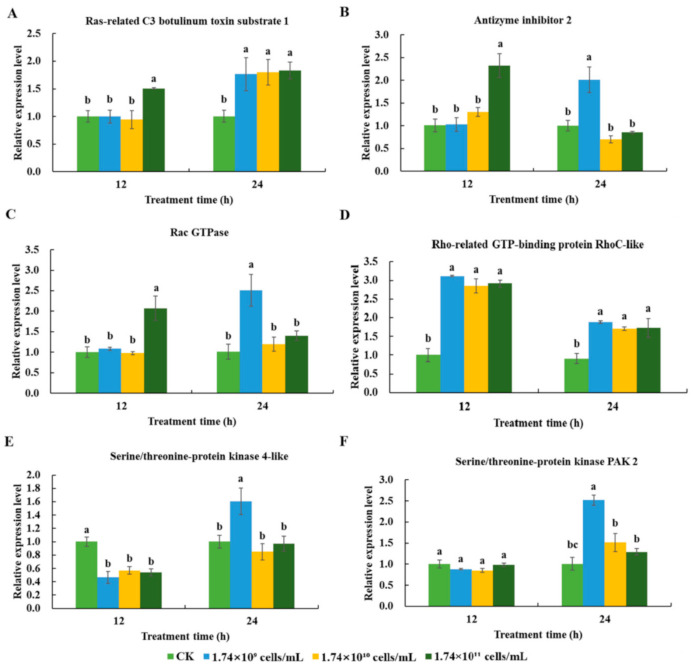
The expression profiling of six cellular immunity- related genes in *R. chinensis* infected with SM1. In the figure, the same lowercase letters indicate that they were not significantly different in gene expression at the same time, but at different treatment concentrations. (**A**) Ras-related C3 botulinum toxin substrate 1; (**B**) Antizyme inhibitor 2; (**C**) Rac GTPase; (**D**) Rho-related GTP-binding protein RhoC-like; (**E**) Serine/threonine-protein kinase 4-like; (**F**) Serine/threonine-protein kinase PAK2.

The expression profiles of six cellular immunity-related differentially expressed genes were analyzed using qRT-PCR. These six genes (Ras-related C3 botulinum toxin substrate 1, rho-related GTP-binding protein RhoC-like, antizyme inhibitor 2, serine/threonine-protein kinase PAK2, Rac GTPase, and serine/threonine-protein kinase 4-like) participated in important cellular immune processes, such as apoptosis, autophagy, and phagocytosis. The results indicated that the expression of these genes was clearly induced by SM1 (Figure 5).

At 12 h, the expression levels of Ras-related C3 botulinum toxin substrate 1 (F = 16.19; df = 3, 8; *p* < 0.01), antizyme inhibitor 2 (F = 34.931; df = 3, 7; *p* < 0.01), and Rac GTPase (F = 39.33; df = 3, 8; *p* < 0.01) were induced only by 1.74 × 10^11^ cells/mL SM1 (Figure 5A–C). The expression level of the rho-related GTP-binding protein RhoC-like (F = 159.63; df = 3, 8; *p* < 0.01) was induced by SM1 (Figure 5D). The expression level of serine/threonine-protein kinase 4-like (F = 25.294; df = 3, 7; *p* < 0.01) was downregulated after infection with SM1 (Figure 5E). For serine/threonine-protein kinase PAK2, there was no difference in expression after infection with SM1 (Figure 5F).

At 24 h, the expression levels of Ras-related C3 botulinum toxin substrate 1 (F = 10.714; df = 3, 8; *p* < 0.01) and rho-related GTP-binding protein RhoC-like (F = 25.061; df = 3, 7; *p* < 0.01) were upregulated by SM1 (Figure 5A,D). The expression levels of serine/threonine-protein kinase 4-like (F = 18.866; df = 3, 8; *p* < 0.01), Rac GTPase (F = 23.833; df = 3, 8; *p* < 0.01), and antizyme inhibitor 2 (F = 29.244; df = 3, 6; *p* < 0.01)) were induced only by 1.74 × 10^9^ cells/mL SM1 (Figure 5B,C,E). The expression of serine/threonine-protein kinase PAK2 (F = 66.133; df = 3, 7; *p* < 0.01) was induced by 1.74 × 10^9^ and 1.74 × 10^10^ cells/mL SM1 (Figure 5F).

#### 3.3.3. Humoral Immune Responses Induced by SM1 in *R. chinensis*

**Figure 6:**
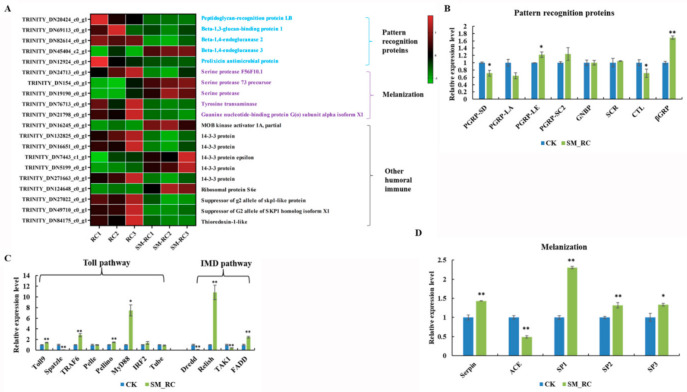
Changes of the humoral immunity-related genes of *R.* *chinensis* after infection with SM1. (**A**) Heatmap displaying the DEGs related to humoral immunity using sequencing data. RC1, RC2, and RC3: The control groups; SM_RC1, SM_RC2, and SM_RC3: *R.* *chinensis* groups infected with SM1. (**B**) Analysis of pattern recognition protein genes expression using qRT-PCR. (**C**) Analysis of the expression of genes related to the Toll and IMD pathways using qRT-PCR. (**D**) Analysis of the expression of genes related to the melanization pathway using qRT-PCR. The star symbols (* *p* < 0.05; ** *p* < 0.01) indicates statistical significance between the treatment and control groups.

Based on the analysis of the sequencing data, a total of 20 humoral immunity-related genes were identified (Figure 6A). They included pattern recognition proteins (PRPs), melanization-related genes, and other humoral immunity-related genes.

To better understand the role of the Toll and IMD pathways in the defense of *R. chinensis* against SM1, we explored the expression patterns of key genes in these pathways in *R. chinensis* by qRT-PCR. Several pattern recognition protein genes were identified in the sequencing data. The expression of peptidoglycan-recognition protein LE (PGRP-LE) and beta-1,3-glucan-binding protein (βGRP) was upregulated in this study. The expression of PGRP-SD and C-type lectin (CTL) was downregulated after *R. chinensis* was infected with SM1 (Figure 6B). For Toll signaling pathway-related genes, the expression of the Toll9, TNF receptor associated factor 6 (TRAF6), Myd88, and Pellino genes was upregulated in *R. chinensis* after infection with SM1, while the expression of Spatzle was downregulated in this study (Figure 6C). For the IMD pathways, the expression levels of the relish and FADD genes were upregulated, and the expression levels of the Dredd and TAK1 genes were downregulated (Figure 6C). These results showed that genes in the Toll and IMD pathways were activated to resist SM1.

The melanization pathway plays an important role in defending against pathogens in insects. Moreover, we measured the expression of genes involved in melanization (Figure 6D). Serine proteases (SPs) are components of the protease cascade of melanization, and these genes can promote the melanization process. The expression levels of SP1, SP2, and SP3 were upregulated in *R. chinensis*. In our study, we found that the expression level of serpin was upregulated, and angiotensin converting enzyme (ACE) was downregulated in *R. chinensis* infected with SM1. Interestingly, the downregulated expression of ACE, which is a negative regulator of this pathway, contributes to the melanization process. According to these research data, we hypothesize that the melanization process in *R. chinensis* was activated by SM1.

## 4. Discussion

Many insects depend on multiple innate immune reactions to eliminate infection by foreign microbes. Clarifying the molecular interaction between microbes and pests would be beneficial for controlling pests. Many studies proved that some immune genes were used as effective RNA interference targets for pest management [26]. Cellular immune and humoral immune responses are two major immune systems in insects that rely on the recognition of pathogenic microbes [27].

The cellular immune response, which mainly includes phagocytosis, encapsulation, and nodulation, is primarily carried out by the hemocyte. In many organisms, endocytosis plays a housekeeping role and prevents the entry of foreign microbes [28]. Autophagy is an important pathway for insects to degrade dysfunctional cell components and eliminate the invaders [29]. We identified some cellular immune pathways from the top 20 enriched KEGG terms, such as endocytosis, lysosome, autophagy, and phagosome (Figure 1C). We attempted to reveal the mechanism underlying the cellular immune response in resisting SM1 using sequencing data. For phagocytosis, cytoplasmic actin can promote the phagocytosis of bacteria as an extracellular immune factor and directly kill the bacteria [30]. Coronin 1 can promote the phagocytosis process by regulating F-actin proteins [31]. The expression of actin and coronin can drive the phagocytosis in insects. In our study, the expression levels of six F-actin and two coronin 1A genes were upregulated in *R. chinensis* infected with SM1 (Figure 4A). V-ATPases are membrane-spanning proton channels in organisms and are important for cellular processes [32,33]. V-ATPases in the plasma membrane are important parts of human disease resistance (for example, cancer), and genetic defects in the human V-ATP enzyme can lead to some diseases [32]. Therefore, V-ATPases can be selected as a potential target for pest management. The expression of V-ATPases was upregulated in this study (Figure 4A), which indicated that V-ATPase might participate in the early and late phagocytosis processes to defend *R. chinensis* against SM1 infection. Autophagy-related genes are conserved in vertebrates and invertebrates [34,35,36]. The autophagy pathway consists of five steps: Initiation, phagophore formation, phagophore elongation, autophagosome fusion with lysosomes, and cargo degradation in the autolysosome [37]. ATG1 is involved in the first steps, and ATG3/ATG8 are involved in the final steps. In our study, ATG1, ATG3, and ATG8 expression was upregulated in *R. chinensis*. These results suggested that *R. chinensis* actively defends against SM1 by autophagy. Endocytosis is one of the important mechanisms of pathogens removal [38,39]. Endocytosis mediates the cellular entry of many viruses, some bacteria, and bacterial toxins [38]. Clathrin is one of the key components of endocytosis and facilitates the process of endocytosis [39]. The expression levels of dynamin genes, which are related to endocytosis, were upregulated in *R. chinensis* infected with SM1, and some genes related to endocytosis were differentially expressed, including dynamins, WASH, WASHC7, UCH8, CHMPs, and VPSs. Many have proven that the host uses endocytosis to clear foreign components [38,39,40,41]. Our data suggested that endocytosis was activated in *R. chinensis* to remove SM1. Multiple endocytic processes, including phagocytosis, endocytosis, and autophagy, fuse with lysosomes to facilitate degradation [42,43,44,45,46]. The expression of several adaptin genes and dipeptidyl peptidase genes, which are related to lysosomes, was upregulated. These results indicated that SM1 might ultimately be affected by the degradation process of cellular immunity in *R. chinensis*. Differential cellular immunity in insects can be induced by bacteria [47,48,49]. This research indicated that the cellular immune response of *R. chinensis* played an essential role in resisting the SM1 infection.

The insect humoral immune system includes the Toll pathway, IMD pathway, and melanization pathway [50]. The Toll and IMD pathways participate in the insect defense response by generating antimicrobial peptides (AMPs). The high expression of AMP genes helps the insect resist infection with foreign microbes [51,52]. Melanization plays a vital role in insects and is activated by bacteria [53]. This process performs multiple functions, such as wound repair and antipathogen activity [54,55]. Humoral immune responses are efficient innate immune systems in insects [26]. The humoral immune response plays an important role in resisting *Bacillus thuringiensis* infection in *P. xylostella* [55]. At present, there is no research regarding the interaction between the humoral immunity of *R. chinensis* and SM1. Humoral immune responses are divided into the Toll pathway, IMD pathway, JAK/STAT pathway, and melanization pathway [56,57,58,59]. In our study, we found 36 differentially expressed genes related to humoral immunity in *R. chinensis* by combining sequencing data and qRT-PCR data. This result indicated that the humoral immune responses of *R. chinensis* might be involved in defending against SM1. PRPs act as the immune receptors that initiate humoral immune responses, and these receptors can activate host immune responses [60]. We only found five differentially expressed PRPs, including PGRP-LB, PGRP-SD, PGRP-LE, CTL, and βGRP. PRPs can increase immune signaling and activate the IMD pathway or Toll pathway [61,62]. These results indicated that the IMD pathway or Toll pathway was activated by SM1 in *R. chinensis*. Subsequently, many key regulator genes of the Toll and IMD pathways, such as Toll9, TRAF6, Myd88, FADD, and relish were identified in *R. chinensis* infected with SM1 using qRT-PCR (Figure 6C). Relish and FADD are important regulator genes in the downstream of the IMD pathway [63,64], and these proteins can transduce the immune signals into the nucleus and promote the production of antimicrobial peptides. TRAF6, Myd88, and Pellino can transduce signals downstream of the Toll pathway and induce effectors [56]. Combined with our results, we hypothesize that the Toll and IMD pathways were activated in *R. chinensis* by SM1. Unfortunately, we could not identify DEGs encoding antimicrobial peptides in our sequencing data. Meanwhile, we could try to identify more antibacterial peptide genes belonging to the Toll and IMD pathways in future research.

Melanization is an essential immune response in insects that functions to eliminate foreign threats [65]. C-type lectins play important roles in melanization, and these lectins can bind invading pathogens and activate melanization in insects [66]. The serine protease cascade and phenoloxidase are actively involved in melanization [67]. In this study, CTL, serine proteases, and serpin were differentially expressed between *R. chinensis* infected with SM1 and control *R. chinensis*. In previous research, ACE was a negative regulator of melanization which can alter the immune response by regulating the activity of PO in locusts [68]. We found that ACE was downregulated in *R. chinensis* infected with SM1 in our study. Melanization is an important innate immune response that functions in killing microbes, wound healing, and accelerating melanotic encapsulation [69,70,71]. Our results indicated that melanization-related genes may participate in resisting against SM1 in *R. chinensis*.

## 5. Conclusions

This study indicated that SM1 infection could affect the cellular and humoral immune responses in *R. chinensis*. Moreover, this study elucidated the mechanism underlying the interaction between *R. chinensis* immunity and SM1, providing a reliable theoretical reference for developing more effective biological control methods. The findings will provide a theoretical foundation for developing immunosuppression in termites.

## Figures and Tables

**Table 1:** Length distribution of *R. chinensis* transcripts.

Transcript Length Interval	Number of Transcripts	Percentage (%)
<500 bp	242,703	61.55
500–1 k	86,025	21.82
1–2 k	40,128	10.18
>2 k	25,434	6.45
Total	394,290	
Length of all transcripts	285,969,777	
N50 (bp)	1088	

## Data Availability

The clean data for the *R. chinensis* and the *R. chinensis* infected by SM1 can be accessed at NCBI with the following accession numbers: SRR18067184, SRR18067185, SRR18067186, SRR18067187, SRR18067188, SRR18067189.

## Supplementary Materials

The following are available online at https://www.mdpi.com/article/10.3390/insects13030226/s1. Figure S1: Number of DEGs assigned to Gene Ontology (GO) categories. Each DEGs was assigned at least one GO term. Figure S2. Functional classification of transcripts into clusters of euKaryotic Ortholog Groups (KOG). Figure S3. Number of unigenes assigned to KEGG Pathway Enrichment. The y-axis indicates the name of the KEGG pathway. Figure S4. Number of DEGs assigned to KEGG Pathway Enrichment. Table S1: Primers used in qRT-PCR; Table S2: Summary of sequencing data analysis; Table S3: Annotation of Nr Transcripts against public databases; Table S4: Differentially expressed immune-related genes in *R. chinensis*.
