# Supplementary material for: Immune Defense Mechanism of Reticulitermes chinensis Snyder (Blattodea: Isoptera) against Serratia marcescens Bizio
Source: Insects. 2022 Feb 24;13(3):226. doi: 10.3390/insects13030226 (PMC8954430; doi:10.3390/insects13030226)
Supplement: Supplementary file 1 [file insects-13-00226-s001.zip › Supplementary Table S2 and Table S3.pdf]

Table S2. Summary of sequencing data analysis.

Sample	Clean Reads Pairs	Clean base(bp)	Length	Q20(%)	Q30(%)	GC (%)
RC1	33,572,113	10,071,633,900	150;150	98.0;97.2	94.1;92.2	42.0;42.0
RC2	32,061,292	9,618,387,600	150;150	98.0;97.1	94.1;91.8	41.9;41.8
RC3	30,728,402	9,218,520,600	150;150	97.9;97.2	93.9;92.2	41.8;41.8
SM-RC1	30,947,431	9,284,229,300	150;150	98.0;97.0	94.2;91.9	42.6;42.6
SM-RC2	30,655,201	9,196,560,300	150;150	97.9;97.2	94.0;92.4	42.8;42.8
SM-RC3	28,939,264	8,681,779,200	150;150	97.9;96.7	94.1;91.2	43.1;43.1

RC1, RC2 and RC3: healthy *R. chinensis* (CK); SM-RC1, SM-RC2 and SM-RC3: *R. chinensis* infected with SM1 (SM-HC); clean read pairs: the data were used for bioinformatic analysis. Q20, Q30: the percentage of bases with a Phred value >20, 30.

Table S3. Annotation of Nr transcripts against public databases.

Database	Number of Transcripts (Percentage)
Total Transcripts	394,290 (100%)
KOG	68,985 (17.50%)
KEGG	78,454 (19.90%)
NR	118,572 (30.07%)
GO	60,043 (15.23%)
Swiss-Prot	101,639 (25.78%)
Unknown	268,243 (68.03%)
