# Supplementary figures and images for: Immune Defense Mechanism of Reticulitermes chinensis Snyder (Blattodea: Isoptera) against Serratia marcescens Bizio
Source: Insects. 2022 Feb 24;13(3):226. doi: 10.3390/insects13030226 (PMC8954430; doi:10.3390/insects13030226)

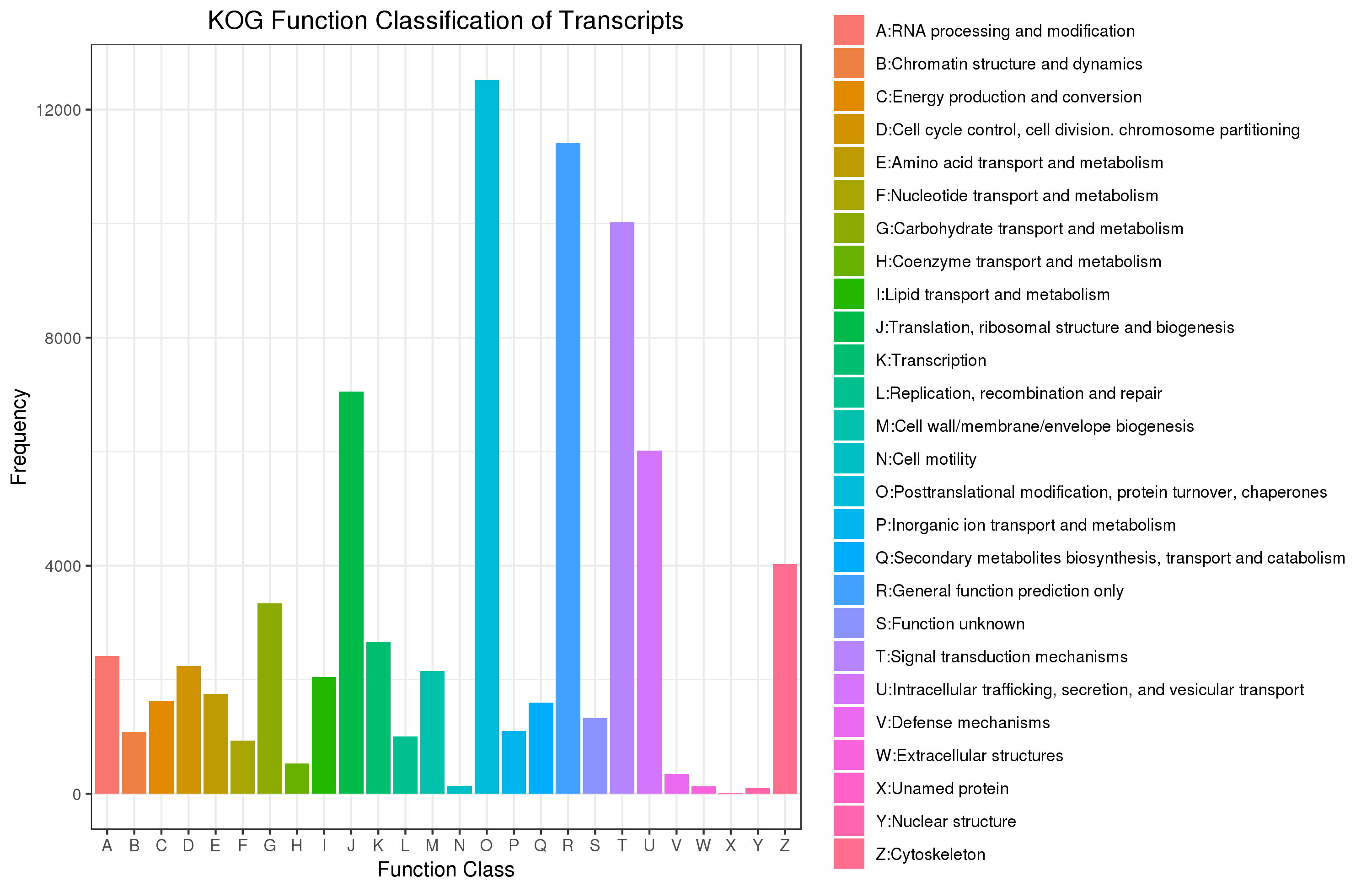

Supplement: Supplementary file 1 [file insects-13-00226-s001.zip › Figure S2.png]

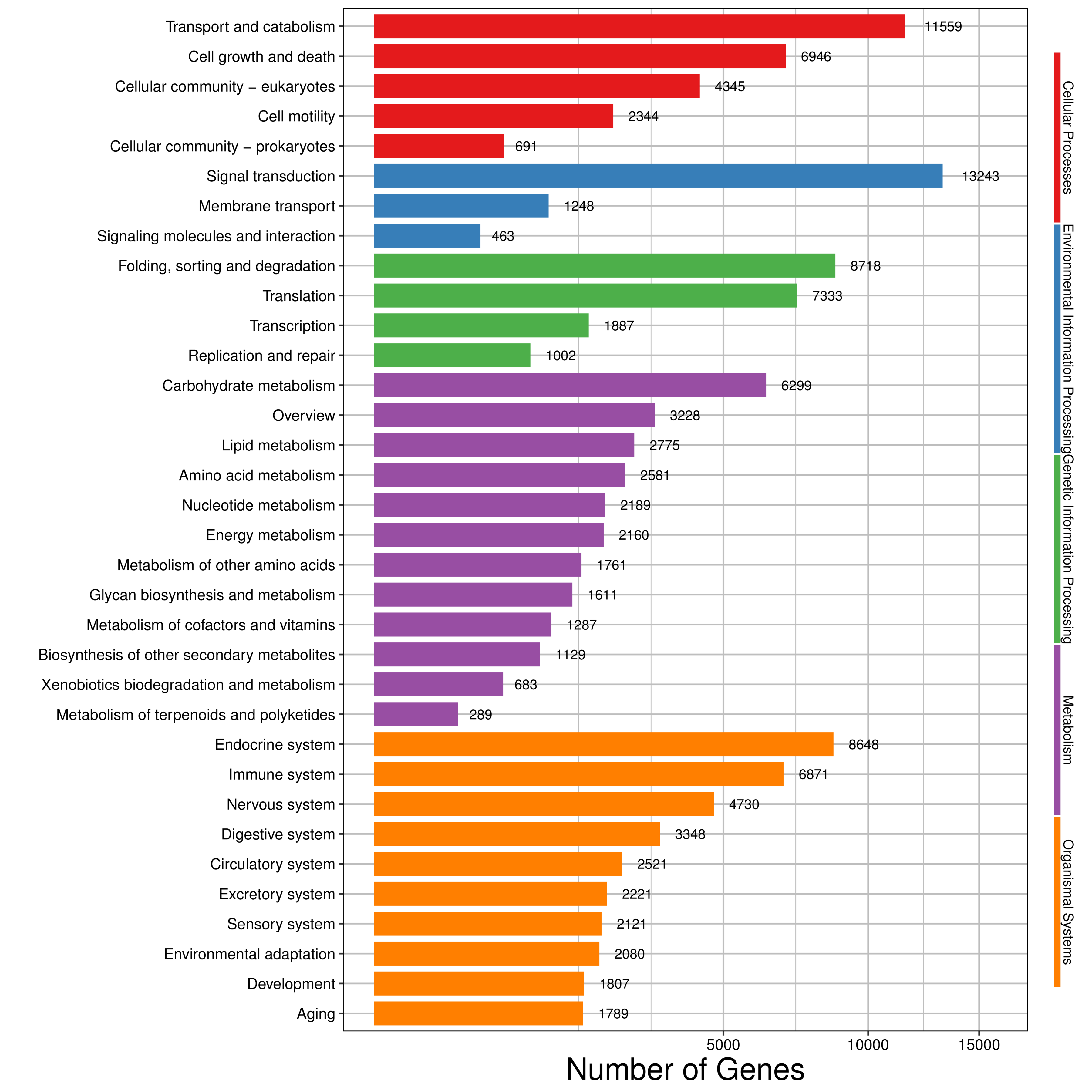

Supplement: Supplementary file 1 [file insects-13-00226-s001.zip › Figure S3.png]

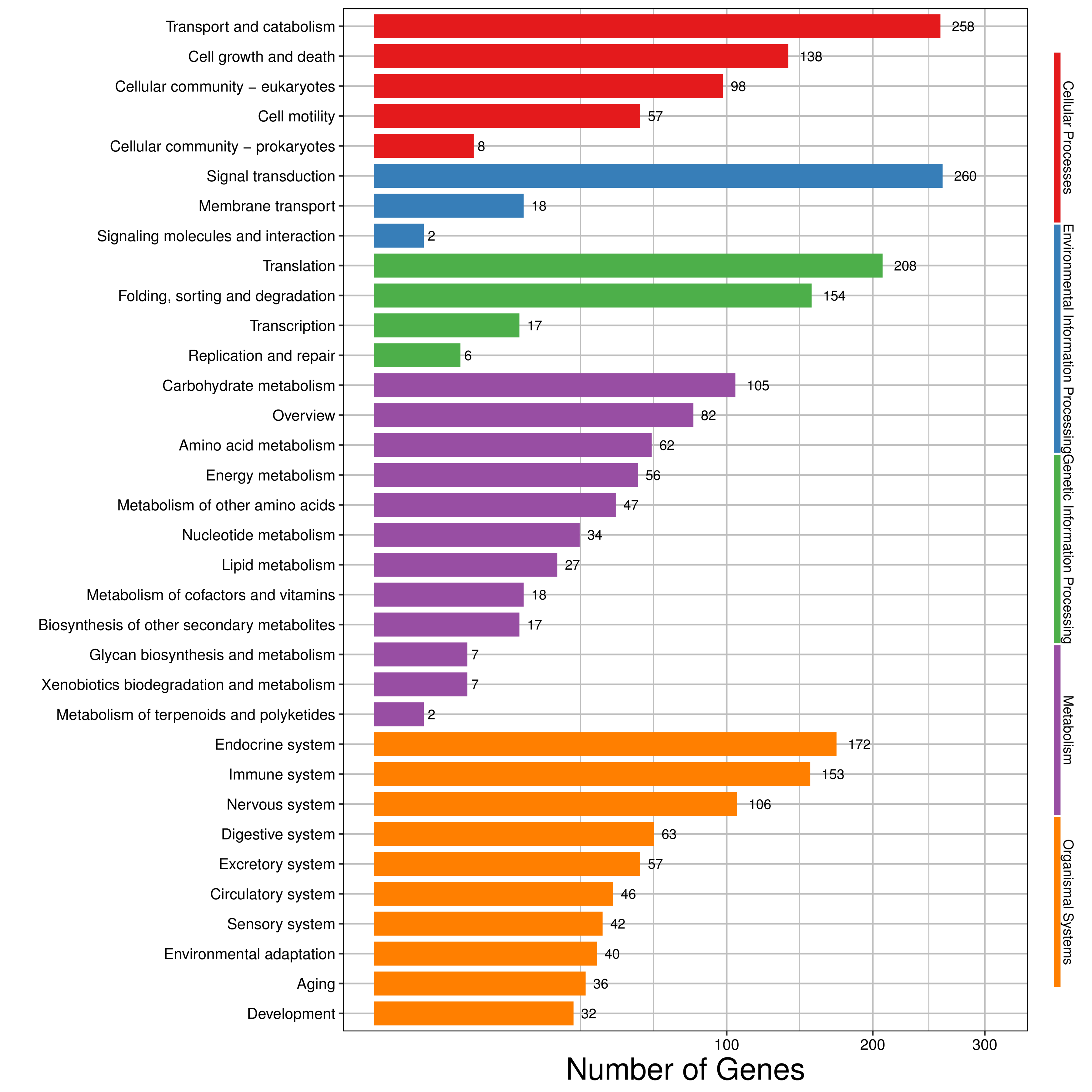

Supplement: Supplementary file 1 [file insects-13-00226-s001.zip › Figure S4.png]
